# Coagulotoxicity of *Bothrops* (Lancehead Pit-Vipers) Venoms from Brazil: Differential Biochemistry and Antivenom Efficacy Resulting from Prey-Driven Venom Variation

**DOI:** 10.3390/toxins10100411

**Published:** 2018-10-11

**Authors:** Leijiane F. Sousa, Christina N. Zdenek, James S. Dobson, Bianca op den Brouw, Francisco C. P. Coimbra, Amber Gillett, Tiago H. M. Del-Rei, Hipócrates de M. Chalkidis, Sávio Sant’Anna, Marisa M. Teixeira-da-Rocha, Kathleen Grego, Silvia R. Travaglia Cardoso, Ana M. Moura da Silva, Bryan G. Fry

**Affiliations:** 1 Laboratório de Imunopatologia, Instituto Butantan, São Paulo 05503-900, Brazil; leijiane.sousa@butantan.gov.br (L.F.S.); tiago.moretto@butantan.gov.br (T.H.M.D.-R.); ana.moura@butantan.gov.br (A.M.M.d.S.); 2 Venom Evolution Lab, School of Biological Sciences, University of Queensland, St. Lucia, QLD 4072, Australia; christinazdenek@gmail.com (C.N.Z.); j.dobson@uq.edu.au (J.S.D.); b.opdenbrouw@uq.edu.au (B.o.d.B.); francisco.cp.coimbra@gmail.com (F.C.P.C.); 3 Fauna Vet Wildlife Consultancy, Glass House Mountains, QLD 4518, Australia; drambergillett@hotmail.com; 4 Laboratório de Pesquisas Zoológicas, Unama Centro Universitário da Amazônia, Pará 68035-110, Brazil; hchalkidis@gmail.com; 5 Laboratório de Herpetologia, Instituto Butantan, São Paulo 05503-900, Brazil; savio.santanna@butantan.gov.br (S.S.); marisa.rocha@butantan.gov.br (M.M.T.-d.-R.); kathleen.grego@butantan.gov.br (K.G.); 6 Museu Biológico, Insituto Butantan, São Paulo 05503-900, Brazil; silvia.cardoso@butantan.gov.br

**Keywords:** venoms, coagulotoxicity, antivenom, variations, adaptive pressures, venom induced consumptive coagulopathy

## Abstract

Lancehead pit-vipers (*Bothrops* genus) are an extremely diverse and medically important group responsible for the greatest number of snakebite envenomations and deaths in South America. *Bothrops atrox* (common lancehead), responsible for majority of snakebites and related deaths within the Brazilian Amazon, is a highly adaptable and widely distributed species, whose venom variability has been related to several factors, including geographical distribution and habitat type. This study examined venoms from four *B. atrox* populations (Belterra and Santarém, PA; Pres. Figueiredo, AM and São Bento, MA)*,* and two additional *Bothrops* species (*B. jararaca* and *B. neuwiedi*) from Southeastern region for their coagulotoxic effects upon different plasmas (human, amphibian, and avian). The results revealed inter– and intraspecific variations in coagulotoxicity, including distinct activities between the three plasmas, with variations in the latter two linked to ecological niche occupied by the snakes. Also examined were the correlated biochemical mechanisms of venom action. Significant variation in the relative reliance upon the cofactors calcium and phospholipid were revealed, and the relative dependency did not significantly correlate with potency. Relative levels of Factor X or prothrombin activating toxins correlated with prey type and prey escape potential. The antivenom was shown to perform better in neutralising prothrombin activation activity than neutralising Factor X activation activity. Thus, the data reveal new information regarding the evolutionary selection pressures shaping snake venom evolution, while also having significant implications for the treatment of the envenomed patient. These results are, therefore, an intersection between evolutionary biology and clinical medicine.

## 1. Introduction

Snakebite is a global crisis due to its neglect relative to other tropical diseases [1]. Snake venoms can attack a myriad of physiological systems, affecting blood coagulation or blood pressure, causing cell death, selectively damaging muscle cells, or interfering with nerve transmission either by preventing nerve signals or preventing nerves from being turned off [2]. However, due to inherent difficulties in working with blood enzyme cascades, research into coagulotoxicity has lagged behind other intensively studied areas such as neurotoxicity. In addition, of the coagulotoxin studies that have been undertaken, only some [3,4,5,6,7,8,9] have included in the experimental design the cofactors calcium and phospholipid, which are necessary to replicate physiological conditions in in vitro assays. Other studies have included only calcium [10,11,12,13,14,15], or neither cofactor [16,17,18], leading to deficiencies in replicating physiological conditions and interfering with accurate interpretation of the effects upon coagulation.

The *Bothrops* genus (lancehead pit-vipers) is an extremely diverse [19] and medically significant snake group, responsible for the majority of envenomations and deaths related to snakebites in South America [20,21]. The main clinical effects of the envenoming by *Bothrops* snakes includes local tissue damage (edema, haemorrhage, and myonecrosis), and systemic effects resulting mainly in blood coagulation disorders [22]. The main protein families involved in systemic coagulopathic effects are snake venom metalloproteinases (SVMPs), snake venom serine proteinase (SVSPs), phospholipases (PLA_2_s) and C-type Lectins (CTLs). These toxins are able to act on components of the coagulation cascade, promote haemorrhage, and interfere with the formation of the haemostatic plug and platelet aggregation [23,24,25,26,27,28,29].

Within the B*othrops* genus, *Bothrops atrox* (common lancehead pit-viper) causes more human fatalities than any other South American snake [20]. *B. atrox* is a highly adaptable species which is widely distributed in a large part of the tropical plains of South America [19] and are also prevalent throughout the Brazilian Amazon. This species is responsible for the most snakebites in the Amazon region [30], and the variability in their venoms has been reported according to ontogeny [31,32], between populations [33,34,35], and within populations [36,37], even extending to the habitat type [38]. Venom variability within *B. atrox* snakes has been related to diverse factors including geographical distribution [33,34,35] and habitat variation [38], which likely reflect variances in prey type and prey escape potential. Venom variability of this species can produce functional diversity able to affect antivenom efficacy, as demonstrated previously by in vitro assays [38,39].

Venom variability associated with functional diversity is particularly critical regarding reactivity with antivenoms, as recently reported in a study involving venoms from *B. atrox* snakes captured in distinct habitats of Western Pará, Brazilian Amazon [38]. This study reported remarkable functional differences in the venom of a *B. atrox* population (from a floodplain habitat), whose high procoagulant activity was not efficiently neutralised by antivenoms. Antivenoms in general are made using an immunising mixture which includes only a limited range of venoms obtained from few species [40]. One complicating matter is that venom composition may vary considerably among different species and, as related above [36,37], between populations within a single species [41]. Therefore, antivenom efficacy may vary considerably due to inter-species and intra-specific variation in venom composition [8].

In Brazil, *Bothrops* antivenom is produced from plasma of horses immunised with a venom mixture of the following species: *Bothrops jararaca* (50%), *Bothrops neuwiedi* (12.5%), *Bothrops alternatus* (12.5%), *Bothrops moojeni* (12.5%), and *Bothrops jararacussu* (12.5%); however, despite its great medical importance, *B. atrox* venom is not included in this immunising mixture.

*Bothrops* species are notable amongst pit-vipers in having the ability to profoundly affect blood chemistry through the procoagulant action of Factor X activation and prothrombin activation [10,39,42,43,44,45,46,47,48,49]. In 1979, Nahas and colleagues undertook a comparative study of 26 *Bothrops* species and reported that prothrombin or Factor X activation functions were present in all *Bothrops* species, in addition to the ability to directly clot fibrinogen, but with high degrees of variability within the genus [50]. More recently, studies about intra and inter-specific variations in *Bothrops* venoms reported differences in procoagulant components between species and within populations of a particular species [39].

It has been demonstrated that SVMPs, particularly of P-III class, are the most abundant within the *Bothrops* genus [51] and also the most antigenic and immunoreactive components in the *Bothrops* venoms, including some *B. atrox* populations from the Brazilian Amazon [33,51]. Consequently, many SVMPs have already been isolated from *Bothrops* venoms, several of which were able to activate coagulation factors, particularly Factor X and prothrombin [47,48,49,52,53,54,55]. Factor V, Factor VII, Factor X, and prothrombin activators have been found in *B. atrox* venoms which were already isolated and characterised, at least partially [42,43,44]. In *B. neuwiedi* venom, Factor X and prothrombin activators have been isolated and characterised [49], and from *B. jararaca* venom a prothrombin activator has also been isolated and characterised [54]. Other characterised toxin types include serine proteases which act in a pseudo-procoagulant manner by cleaving fibrinogen to form transient, weak fibrin clots, and also serine proteases which generate endogenous thrombin [56,57,58].

Despite the aforementioned studies on coagulotoxins within *Bothrops* venoms, little has been investigated regarding evolutionary variations in the basic underlying biochemistries, such as relative dependence on the calcium or phospholipid cofactors, and how this influences function. In addition, a more systematic investigation has not been undertaken to evaluate the *Bothrops* antivenom efficacy against procoagulant mechanisms of Factor X activation and prothrombin activation. Another important under-investigated issue which concerns the ecology of snakes is the relative venom potency of different *Bothrops* species and populations on taxon-specific plasma. Most studies to date have tested venoms against only one type of plasma, usually from humans or rodents [45,51]. The sole study in this regard showed that purified SVMPs, isolated from *B. neuwiedi* venom, differentially affected human, rat, and chicken plasmas [49].

Therefore, in order to fill the aforementioned knowledge gaps, this study examined venoms of four populations of *B. atrox* snakes, captured from different habitat types within the Brazilian Amazon, in addition to two other *Bothrops* species, *B. jararaca* and *B. neuwiedi*, whose venoms are used for the production of the Instituto Butantan *Bothrops* antivenom. These venoms were submitted to a battery of analyses to provide a comprehensive examination of the venom effects: (i) relative coagulotoxicity; (ii) relative dependence of venoms on cofactors (calcium and phospholipid); (iii) relative efficacy of the *Bothrops* antivenoms against *Bothrops* venoms; and (iv) taxon-specific effects on amphibian, avian, and human plasma. The results obtained in this study advance our understanding of evolutionary selection pressures, serum therapy for human envenomations, and the fundamental biochemistry underpinning venom variation.

## 2. Results

### 2.1. Coagulation Analyses Using a Stago STA-R Max Coagulation Analysis Robot

#### 2.1.1. Plasma Clotting Activity

**Figure 1:**
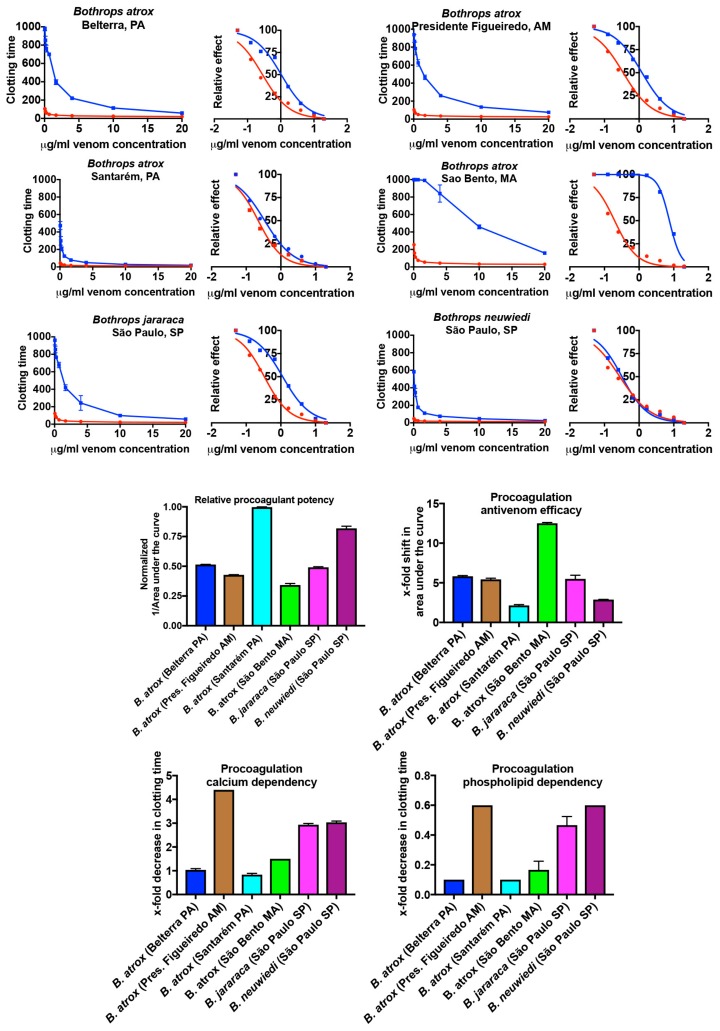
Variation in clotting speed, antivenom efficacy, and venom biochemistry measured on a Stago STA-R Max coagulation analyser. 8-point dilutions are shown in curve and logarithmic presentations. Venom is in red line, venom + antivenom in blue line. Relative procoagulant potency is visualised by calculating 1/AUC, with AUC = area under the curve. Antivenom efficacy results are shift in AUC; if no shift occurred, this would have a value of 0. Therefore, larger numbers are indicative of higher relative antivenom efficacy. Cofactor dependency studies: larger numbers indicate greater dependency, if there was no change with or without a cofactor this would have a value of 0. Data points are *n* = 3 means and standard deviations. Note for many data points in the curves the error bars are smaller than the line icons.

The venoms showed appreciable variation in their clotting times at the 20 μg/mL concentration, with the rank order from most to least potent, in seconds until clot formed, as follows: *B. atrox* (Santarém, PA) 11.1 ± 0.05; *B. neuwiedi* (São Paulo, SP) 13.5 ± 0.7; *B. atrox* (Belterra, PA) 20.3 ± 0.2; *B. jararaca* (São Paulo, SP) 21.2 ± 0.7; *B. atrox* (Pres. Figueiredo, AM) 25.5 ± 0.3; and *B. atrox* (São Bento MA) 30.1 ± 6.1. In order to further elucidate greater resolution of the relative procoagulant activity of the venoms, 8-point dilution curves were conducted (20, 10, 4, 1.667, 0.667, 0.25, 0.125, and 0.05 μg/mL). Significant variation was again evident in the procoagulant toxicity, with the Santarém population again considerably more toxic than the other *B. atrox* populations, in addition to *B. jararaca*, and *B. neuwiedi*, as demonstrated by having a much smaller area under the curve (Figure 1). The rank order (from most to least potent) for the dilution curves was the same as the rank order for the 20 μg/mL concentrations: *B. atrox* (Santarém, PA) 272.4 ± 0.8; *B. neuwiedi* (São Paulo, SP) 331.8 ± 7.4; *B. atrox* (Belterra, PA) 527.5 ± 1.2; *B. jararaca* (São Paulo, SP) 553.2 ± 6.3; *B. atrox* (Pres. Figueiredo, AM) 635.2 ± 3.4; and *B. atrox* (São Bento, MA) 794.8 ± 31.7. The maximum clotting time at the highest venom concentration and area under the dilution curves had a correlation of 0.997.

Antivenom efficacy (calculated by dividing the area under the curve for the antivenom dilution series by the area under the curve for the dilution series without antivenom) was also variable (Figure 1). The antivenom efficacy (presented as an x-fold shift in the area under the curve, whereby no shift would have a value of 0) rank order (from best to poorest neutralised) was: *B. atrox* (São Bento, MA) 12.5 ± 0.1; *B. atrox* (Belterra, PA) 5.8 ± 0.1; *B. jararaca* (São Paulo, SP) 5.5 ± 0.5; *B. atrox* (Pres. Figueiredo, AM) 5.4 ± 0.2; *B. neuwiedi* (São Paulo, SP) 2.9 ± 0.1; and *B. atrox* (Santarém PA) 2.1 ± 0.1. There was an inverse correlation between potency and antivenom efficacy (*−*0.917), whereby the most potent venoms were the least neutralised.

The venoms also differed in the basic biochemistry underpinning the procoagulant activity (Figure 1). The calcium dependency (calculated as dividing the 20 μg/mL clotting time (with both cofactors in the assay) by the 20 μg/mL clotting time with only phospholipid in the assay and then subtracting 1, such that a value of zero would indicate no dependency) rank order (from most dependent to least dependent) was: *B. atrox* (Pres. Figueiredo, AM) 4.4 ± 0.1; *B. neuwiedi* (São Paulo, SP) 3.0 ± 0.1; *B. jararaca* (São Paulo, SP) 2.9 ± 0.1; *B. atrox* (São Bento, MA) 1.5 ± 0.1; *B. atrox* (Belterra, PA) 1.04 ± 0.1; *B. atrox* (Santarém, PA) 0.8 ± 0.1. The phospholipid dependency (calculated as dividing the 20 μg/mL clotting time (with both cofactors in the assay) by the 20 μg/mL clotting time with only calcium in the assay and then subtracting 1, such that a value of zero would indicate no dependency) rank order was: *B. atrox* (Pres. Figueiredo, AM) 0.6 ± 0.001; *B. neuwiedi* (São Paulo, SP) 0.6 ± 0.001; *B. jararaca* (São Paulo, SP) 0.5 ± 0.05; *B. atrox* (São Bento, MA) 0.2 ± 0.05; and *B. atrox* (Belterra, PA) 0.1 ± 0.001 = *B. atrox* (Santarém PA) 0.1 ± 0.001. 

#### 2.1.2. Factor X and Prothrombin Activation Activity

**Figure 2:**
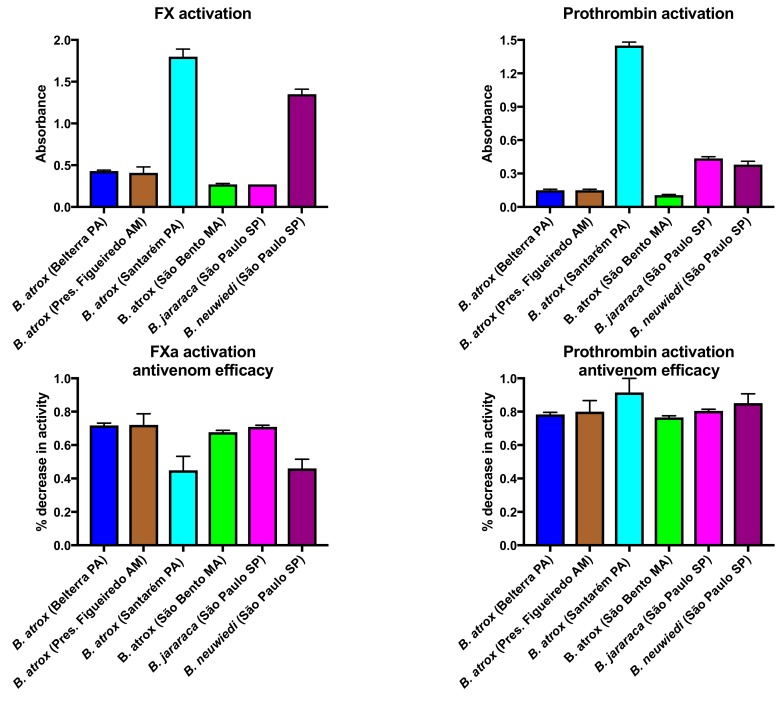
Variation in the ability to activate the clotting zymogens Factor X and prothrombin, measured on a Stago STA-R Max coagulation analyser. Activation studies: larger numbers indicate greater activity. Antivenom assays: larger numbers indicate greater antivenom efficacy. Data points are *n* = 3 means and standard deviations.

In order to ascertain relative activation of the zymogens Factor X and prothrombin, additional tests were undertaken, including determining the relative efficacy of antivenom upon each activity for each venom. Factor X activation activity was significantly correlated with procoagulant activity (0.889) and prothrombin activation activity was also significantly correlated with procoagulant activity (0.742), and they were strongly correlated with each other (0.824). Significant variation between venoms was evident in the activation of each zymogen (Figure 2), with the Santarém population considerably more effective than the other *B. atrox* populations in activating both Factor X and also prothrombin. The Factor X activation rank order (from most to least potent) was: *B. atrox* (Santarém, PA) 1.8 ± 0.09; *B. neuwiedi* (São Paulo, SP) 1.3 ± 0.05; *B. atrox* (Pres. Figueiredo, AM) 0.41 ± 0.07; *B. atrox* (Belterra, PA) 0.43 ± 0.01; *B. jararaca* (São Paulo SP) 0.27 ± 0.01; = *B. atrox* (São Bento, MA) 0.27 ± 0.01. The prothrombin activation rank order (from most to least potent) was: *B. atrox* (Santarém, PA) 1.45 ± 0.03; *B. jararaca* (São Paulo, SP) 0.44 ± 0.02; *B. neuwiedi* (São Paulo, SP) 0.38 ± 0.03; *B. atrox* (Pres. Figueiredo, AM); 0.15 ± 0.01; = *B. atrox* (Belterra, PA) 0.15 ± 0.01; *B. atrox* (São Bento, MA) 0.11 ± 0.01. *B. atrox* (Santarém PA) much stronger than all other venoms in both activities. 

The antivenom was able to neutralise both activities but was much more effective in neutralising the prothrombin activation activity than the Factor X activation activity (Figure 2). The antivenom was particularly less effective in neutralising the Factor X activation activity of both *B. atrox* (Santarém, PA) and *B. neuwiedi* relative to the neutralisation of the prothrombin activation by the same species. These two venoms, *B. atrox* (Santarém) and *B. neuwiedi*, were also the most potently procoagulant, and the least neutralised by antivenom in the plasma tests. There was, thus, an inverse relationship between antivenom neutralisation of the plasma and the relative contribution of Factor X activation activity contribution to procoagulation, whereby the venoms with the highest degree of Factor X activation were the least neutralised. Prothrombin activation, however, was neutralised well for all species. 

### 2.2. Thromboelastography Analyses Using Haemonetics TEG5000s

Thromboelastographic assays were undertaken using human, chicken and toad plasmas, to characterise the main clot parameters in order to investigate possible selectivity of venom for a specific type of plasma, in addition to tests on purified human fibrinogen.

#### 2.2.1. Thromboelastography Tests Using Plasma

**Figure 3:**
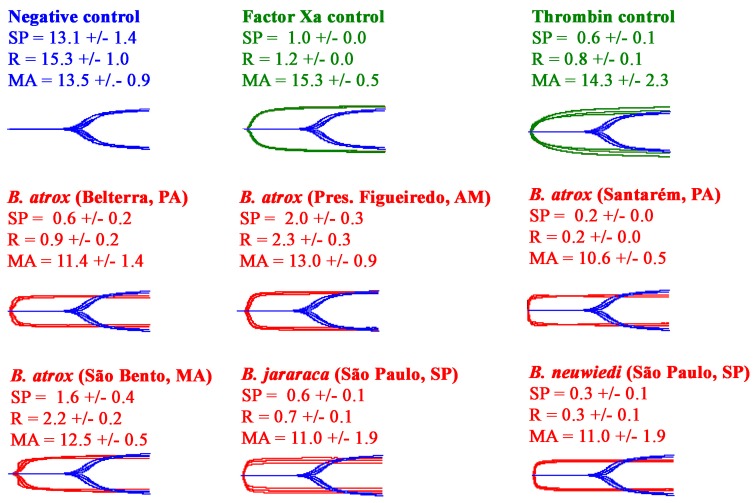
Variations in the ability of venoms to clot human plasma, measured on Haemonetics TEG5000 Thromboelastography analysers for 30 min. For visualisation purposes, the Factor Xa control, thrombin control, and venom experimental traces are overlaid with the spontaneous clotting negative control. All traces are *n* = 3. Values are *n* = 3 means and standard deviation. SP = split point—the time at which clot formation starts; R = time to reach 2 mm amplitude; MA = maximum amplitude.

**Figure 4:**
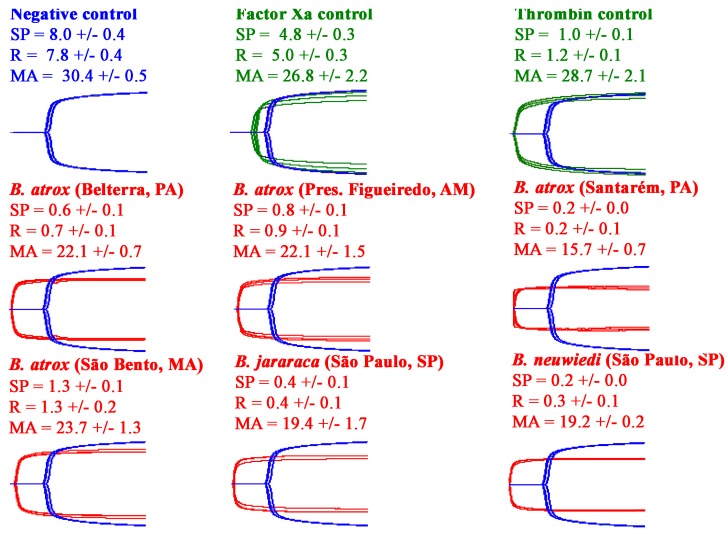
Variations in the ability of venoms to clot avian plasma, measured on Haemonetics TEG5000 Thromboelastography analysers for 30 min. For visualisation purposes, the Factor Xa control, thrombin control, and venom experimental traces are overlaid with the spontaneous clotting negative control. All traces are *n* = 3. Values are *n* = 3 means and standard deviation. SP = split point—the time at which clot formation starts; R = time to reach 2 mm amplitude; MA = maximum amplitude.

**Figure 5:**
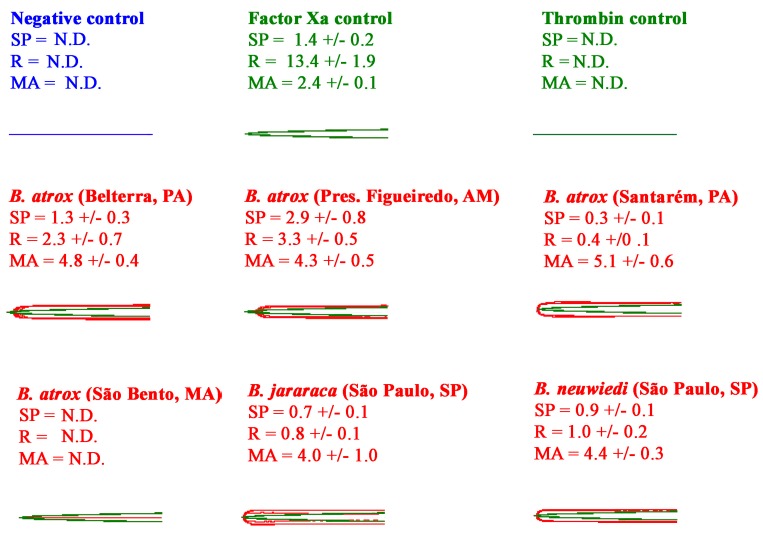
Variations in the ability of venoms to clot amphibian plasma, measured on Haemonetics TEG5000 Thromboelastography analysers for 30 min. For visualisation purposes, the venom experimental traces are overlaid with the Factor Xa control. All traces are *n* = 3. Values are *n* = 3 means and standard deviation. SP = Split Point- the time at which clot formation starts; R = time to reach 2 mm amplitude; MA = maximum amplitude. N.D. = not detectable.

All the venoms showed the ability to rapidly clot human plasma to form strong, stable clots (Figure 3). Testing on avian (Figure 4) and amphibian (Figure 5) plasmas, however, revealed a differential activity. While the avian clotting patterns was congruent with that upon human plasma, the amphibian plasma was discordant in this regard. This lack of congruence was largely due to the inactivity of *B. atrox* (São Bento, MA) on amphibian plasma. However, unlike the tests upon human and amphibian plasmas, none of the venoms were able to form clots in the avian plasma of the same strength (MA) as the controls. Further, despite being the fastest venom (SP values in Figure 4), the *B. atrox* (Santarém, PA) venom formed significantly weaker clots in the avian plasma (*p* = 0.02 for *B. neuwiedi,* the closest other venom) than the other venoms in that same plasma (MA values in Figure 4). Also notable in the TEG analyses was that, unlike the avian and human plasmas, the toad plasma did not spontaneously form a clot after being recalcified.

#### 2.2.2. Thromboelastography Tests Using Fibrinogen

**Figure 6:**
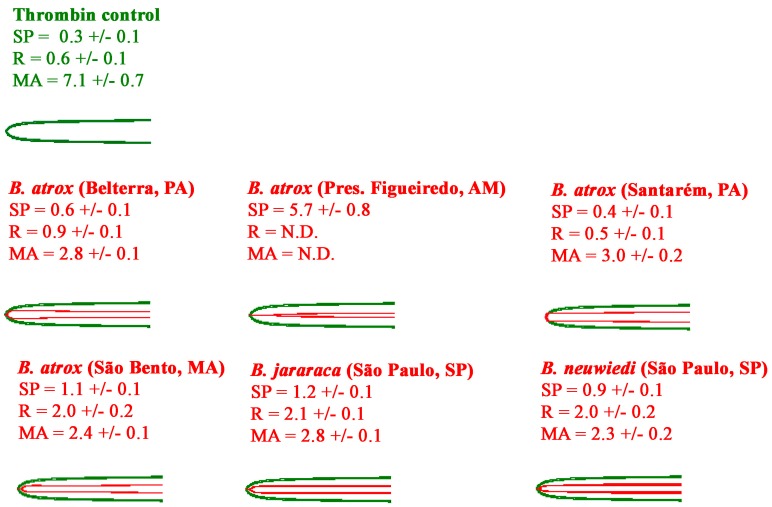
Variations in the ability of venoms to clot human fibrinogen, measured on Haemonetics TEG5000 thromboelastography analysers for 30 min. For visualisation purposes, the venom experimental traces are overlaid with the thrombin control. All traces are *n* = 3. Values are *n* = 3 means and standard deviation. SP = Split Point- the time at which clot formation starts; R = time to reach 2 mm amplitude; MA = maximum amplitude.

In order to ascertain the retention of the basal coagulotoxic action of fibrinogenolysis, additional thromboelastography analyses were under taken using human fibrinogen in place of plasma. All venoms showed the ability to rapidly form weak, pseudo-procoagulant clots when tested directly on fibrinogen, with the exception of *B. atrox* (Pres. Figueiredo AM). This venom was notably weaker and slower in this activity than the other venoms, with the R and MA not detectable, as they were below the machine measurement threshhold (Figure 6).

### 2.3. Fibrinogen Gel Analyses

**Figure 7:**
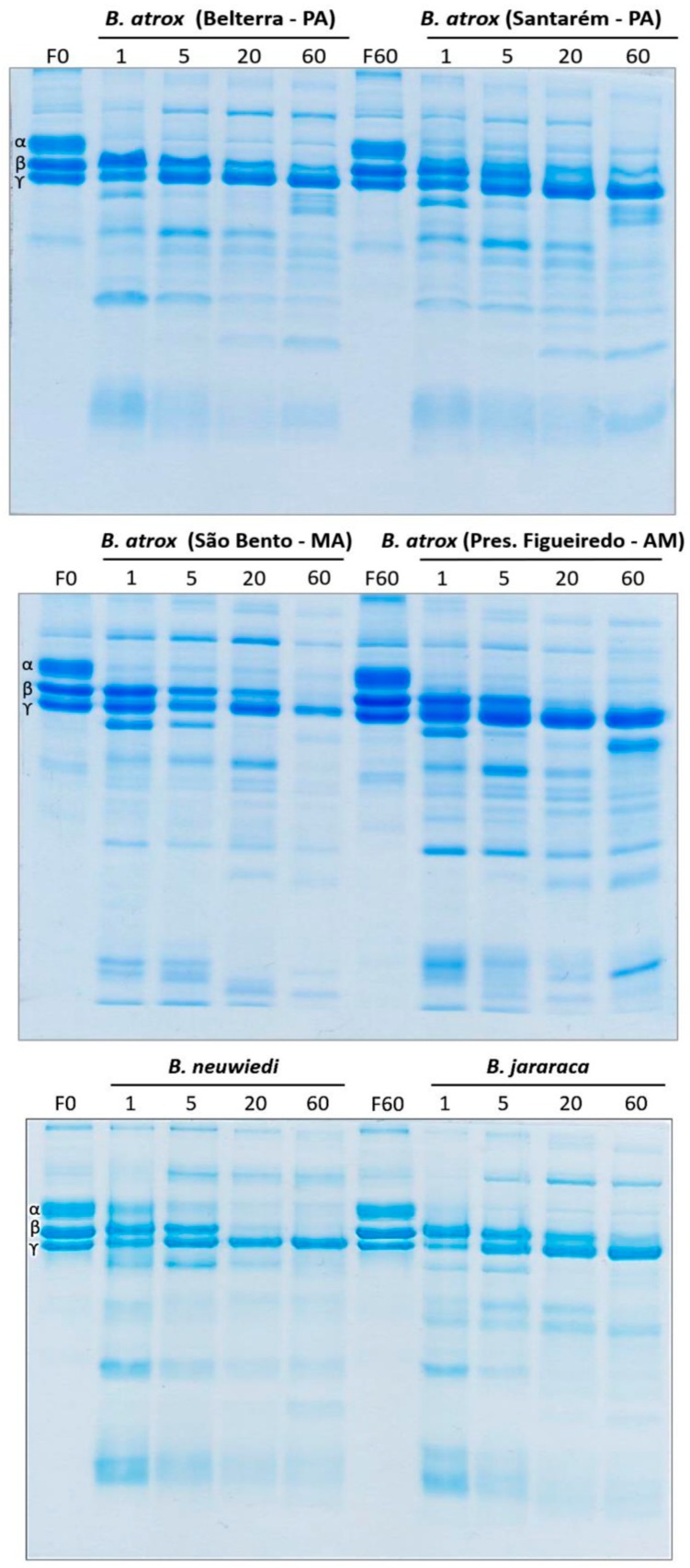
1D SDS-PAGE visualisation of fibrinogenolytic effects. F0 = time 0 min fibrinogen control; F60 = time 60 min fibrinogen control; 1, 5, 20, and 60 = experimental time periods. Each condition was run in triplicate. Representative gels are shown.

**Figure 8:**
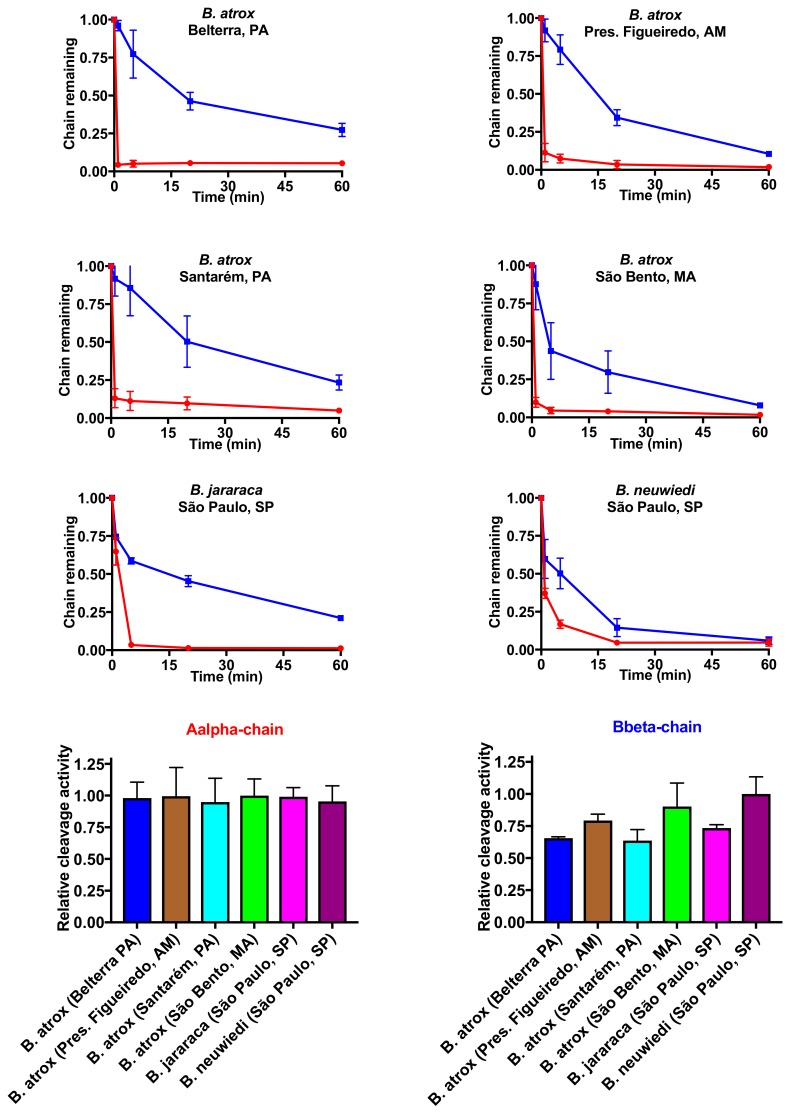
Differential effect upon Aalpha and Bbeta fibrinogen chains. Data points are *n* = 3 means and standard deviations.

Next, in order to quantify fibrinogen chain cleavage specificity and relative rate of action, additional fibrinogen assays were undertaken whereby venom was incubated with fibrinogen for varying periods of time and then visualised using 1D SDS-PAGE gels (Figure 7), followed by quantification of band densities (Figure 8). All venoms were faster to act—and more potent—upon the Aalpha fibrinogen chain than the Bbeta chain and with greater variability in action seen for the Bbeta chain relative to the highly conserved action on the Aalpha chain.

## 3. Discussion

We investigated differences in the venom biochemistry influencing procoagulant action and reactivity with *Bothrops* antivenom in *B. atrox* populations from different locations in the Brazilian Amazon (Santarém and Belterra–PA, São Bento–MA, Pres. Figueiredo–AM) in addition two other *Bothrops* species (*B. jararaca* and *B. neuwiedi*) of great medical importance in Brazil. This study documented significant variation not only in procoagulant potency of the venoms between the three species (*B. atrox, B. jararaca*, and *B. neuwiedi*), but also revealed previously undocumented intraspecific variation between populations of *B. atrox* from the Brazilian Amazon (Figure 1). Geographical variations in clotting and fibrinolytic activities of *B. atrox* venoms from Venezuela have been reported [35], where significant differences in clotting speed were observed, but cofactor dependency was not investigated, which prevents a comparison with our results. In addition, these authors also did not evaluate antivenom efficacy against these activities.

All *Bothrops* species and populations in the present study shared the derived procoagulant activities that makes this genus unique amongst American pit-vipers. These venoms generate endogenous thrombin through the activation of Factor X and prothrombin (Figure 2), resulting in strong, stable fibrin clots in the plasma. Thromboelastography comparison of derived procoagulant action on plasma due to the Factor X and prothrombin activation functions (Figure 3), in comparison to the ancestral pseudo-procoagulant action upon fibrinogen to directly form weak, unstable fibrin clots (Figure 7 and Figure 8), indicates that the derived action thrombin generating function is faster and stronger, and therefore would be the driving force in prey subjugation. However, the fact that all *Bothrops* species and *B. atrox* populations had both procoagulant and pseudo-procoagulant functions is consistent with the complex coagulopathic effects produced in human envenomation by *Bothrops* snakes [22]. 

In human bite victims, the dilution of the venom into such a large blood volume would result in venom-induced consumption coagulopathy due to the procoagulant toxin generation of endogenous thrombin, which consumes the clotting factors, resulting in a disappearance of fibrinogen and a net anticoagulant state [59]. In such a scenario, unlike prey items which rapidly succumb to the venom, human victims would survive long enough for the pseudo-procoagulant actions to form weak, transient clots with remaining fibrinogen, thereby potentiating the net anticoagulation effects. These weak clots were demonstrated by our thromboelastographic analyses using human fibrinogen instead of plasma for all venoms analysed in this study, with particularly weak clots formed by the *B. atrox* venom from the Pres. Figueiredo population (Figure 6). In fibrinogen gel analyses, the venoms each showed similar cleavage profiles for Aalpha chain, slightly variation upon the Bbeta, and no effects on gamma chain. Pseudo-procoagulant activity on fibrinogen has been associated with thrombin-like enzymes (SVSPs), while non-clotting fibrinogenolytic properties can be related to both thrombin-like enzymes and fibrinogenolytic SVMPs [60]. Most SVMPs with fibrinogenolytic activity cleave Aalpha and Bbeta chains with preference for the former [61]. 

Of particular concern in our study is that the ability of the antivenom to neutralise the procoagulant activity of these medically important snakes varied substantially. This was especially the case for the extremely potent *B. atrox* Santarém population, which was not only the most potent but also the least neutralised (Figure 1). *B. atrox* venom from Santarém population was obtained from snakes collected in a floodplain (várzea) habitat, and these findings corroborate previous results reported by Sousa and colleagues showing a low neutralisation of the procoagulant activity of this venom by *Bothrops* antivenom [38]. The *B. neuwiedi* venom was nearly as potent as the *B. atrox* Santarém population and was similarly not well neutralised by the antivenom. In contrast, *B. jararaca* venom and the *B. atrox* populations (Belterra-PA, Pres. Figueiredo-AM and São Bento-MA) were neutralised with greater efficacy (Figure 1). These findings are surprising, considering that *B. jararaca* and *B. neuwiedi* venoms are included in the immunising mixture used to prepare the antivenom. *B. jararaca* makes up 50% of the immunisation mixture, and was well neutralised. In contrast, *B. neuwiedi* represents 12.5% of the immunising mixture, it was poorly neutralised. However, this does not seem to be a simple quantitative matter, since the antivenom also showed a high efficacy against the venoms of three of the four *B. atrox* populations, despite the venom of this species not being used in the immunising mixture. 

The lowest relative efficacy of the antivenom against the procoagulant activity induced by venoms of *B. atrox* from Santarém and *B. neuwiedi* correlates with a higher amount of Factor X activating toxins in these two venoms (Figure 2). In contrast, the *Bothrops* antivenom was very efficient in preventing prothrombin activation by all venoms. These results thus suggest that the antivenom performs better in neutralising prothrombin activating toxins than Factor X activating toxins. The poor antivenom plasma performance for *B. atrox* (Santarém) and *B. neuwiedi* thus appears to stem from poorly-neutralised Factor X activation activity for these two species, while the prothrombin activation activity was well neutralised for both species. In contrast, the other species did not display such significant variation in neutralisation by antivenom for Factor X versus prothrombin activation activity. This suggests the surface chemistry and geometry for the Factor X activating toxins in the *B. atrox* (Santarém) and *B. neuwiedi* venoms differ from that of the other venom studied here, and that these variations influence antivenom epitope-paratope interations.

Our results not only reveal that *Bothrops* venoms are extremely potently procoagulant, but that there is significant variation in dependence of the venoms on the cofactors calcium and phospholipid, with relative reliance not correlated with overall potency (Figure 1) or relative Factor X or prothrombin activation activity (Figure 2). During plasma collection, calcium is deliberately stripped out of the plasma through the use of citrate as a preservative in order to prevent spontaneous coagulation. Therefore, the replenishment of calcium to the plasma is necessary to replicate physiological conditions. In addition, various steps of the coagulation cascade involve enzyme cleavage of zymogens (proenzymes) to generate thrombin, with most of these cleavage steps occurring on negatively charged phospholipid membrane surfaces, with the requirement of calcium ions [3,62]. Activated platelets are the major source of phospholipids in the blood, therefore laboratory tests using platelet poor plasma are deficient in a source of phospholipid. While the venoms were still active, to a degree, in the absence of one or both cofactors, the relative activity was reduced, and the decrease in activity varied between venoms for both cofactors. Therefore, conducting tests in the presence of both cofactors is essential to properly ascertain activity and therefore determine the real-world efficacy of antivenoms. Previous work on crude venoms and purified fractions have in some cases conducted assays with and without one or both cofactors [48,53,55,63], but did not present relative activities for the cofactor presence or absence and, therefore, those prior studies cannot be directly compared to our results. Other studies on *Bothrops* crude venoms or purified fractions did not include either cofactor, which likely resulted in reportedly lower toxicities than noted here [18,42,43,44,54]. One study which investigated calcium dependency for *B. asper* venoms from Mexico determined that those venoms were not calcium-dependent [10].

One interesting aspect for a biological interpretation of the data is that most of the studies related to procoagulant activity of *Bothrops* venoms have been performed with human plasma and interpreted according to human envenomings. However, it has been accepted that procoagulant activity is a fundamental feature of pit-viper venoms for the purpose of prey capture. In prey animals, the rapid formation of endogenous thrombin by *Bothrops* venom could result in prey incapacitation through stroke induction, with this function convergent with not only other vipers such a *Echis* [8], but diverse snakes from other families such as *Dispholidus* and *Thelotornis* with the Colubridae [9], *Hoplocephalus, Notechis, Paroplocephalus,* and *Tropidechis* within the Elapidae [5], and *Atractaspis* within the Lamprophiidae [6]. In addition, snake venom variability has been correlated to differences in the diet of snakes from different geographical regions [64]. 

In consideration of the importance of procoagulant effects of the venom to *Bothrops* snakes to subjugate prey items, thromboelatographic analyses were undertaken to investigate the main clot parameters in animal plasmas as models of amphibian (cane toad) and avian (chicken) potential prey types in comparison to the effects on human plasma. This study revealed taxon-specific venom effects, which could be consistent with the evolutionary history of the *B. atrox* populations regarding selection pressure of prey availability and prey escape. This was particularly evident for the *B. atrox* Santarém population, which occupies a floodplain ecological niche, and which annually alternates between periods of drought and flood [38]. The dry season could accommodate a great taxonomical diversity of available prey items, but with reduced prey availability, while during the flood non-amphibian prey may be very scarce and amphibians plentiful [38]. In addition to seasonal variability in prey diversity and availability, there is also a seasonal variation in prey escape potential and a differential ability to track prey, with amphibian prey items during the flood season having highest degree of prey escape potential and lowest prey-tracking potential. Thus, the *B. atrox* Santarém population has the seasonally highest variation in prey diversity and availability, and highest prey escape potential; therefore there is a strong evolutionary selection pressure not only for a complex venom active against a wide diversity of taxon-groups, but also for a fast acting venom capable of rapid prey immobilisation. This is consistent with the *B. atrox* (Santarém) venom being the fastest acting and most potent against all plasma types tested here (Figure 1, Figure 4 and Figure 5). This is consistent with other studies that have shown that higher prey escape potential selects for faster acting venom [41,65]. Thus while the *B. atrox* Santarém population formed slightly weaker clots on the avian plasma than the venoms from the others populations, it was the fastest acting venom on avian plasma and all other plasmas. This result suggests that the procoagulant activity in the venom of the Santarém population has an important role in the subjugation of avian prey, despite the inducement of slightly weaker clots on avian plasma.

Thus, there appears to be a trade-off for speed of action relative to the strength of the clot. The activation of clotting factors is a *de novo* activity for the procoagulant enzymes in *Bothrops* venom. Neofunctionalisation of serine proteases and metalloproteases for activation of clotting factors, such as Factor X and prothrombin, are key evolutionary events underpinning the evolutionary success of the *Bothrops* genus. This is in comparison to the prothrombin activation by Australian elapid venoms which have in the venom activated Factor X, which evolved at the base of the Australian snake radiation, and in the *Oxyuranus/Pseudonaja* clade, the secondary inclusion of Factor Va [66,67,68]. Thus in the Australian elapids there are structural limitations imposed on the venom enzymes due to this historical contingency, which limits their adaptive flexibility. This is reflective of a fundamental mode of venom evolution, in which venom glands promiscuously express in the glands proteins which have important endogenous functions in other tissues [69]. In contrast, since the thrombin generating enzymes in *Bothrops* are ancestrally non-clotting enzyme repurposed for a derived clotting function, they have greater adaptive flexibility.

Therefore, a different sort of selection pressure is operating on repurposed enzymes, such as in *Bothrops*, than would be the case for the use of endogenous blood clotting factors as venoms components such as in Australian elapids. This adaptive flexibility allows for structure-function evolutions that trade-off of lower efficiency of action for greater speed of action. Thus, the newly evolved, venom-specific, cleavage site in prothrombin may be different than the cleavage site by the endogenous prothrombinase complex. For the venom forms, the selection pressure is speed of action, to produce a stroke inducing blood clot which, therefore, does not have to be as well-ordered or stable as the blood clot produced during normal blood clotting. In contrast, for endogenous blood clotting, the selection pressure is for reliable, reproducible formation of stable clots. As the venom enzymes are repurposed enzymes that have a non-clotting basal function, they are free of the structural constraints imposed upon the Factor Xa:Factor Va prothrombinase complex, which has been shown to be a limiting factor for elapid venoms which use Factor Xa as venom component [10]. Consequently, the *Bothrops* venom enzymes may have evolved the ability to activate Factor X and prothrombin using cleavage sites distinct from those of the natural clotting cascade activator.

The unnatural activation of Factor X and prothrombin at cleavage sites upstream or downstream of the endogenous cleavage site may produce aberrantly active forms of the endogenous clotting factors FXa or thrombin, which in turn may produce aberrant product when cleaving the next protein in the clotting cascade, with the cumulative effect of aberrant thrombin cleaving fibrinogen in an aberrant manner to produce aberrant fibrin clots. For normal blood clotting, such aberrant fibrin clots may have catastrophic outcomes, but for the snake venoms the effect is neutral as the selection pressure is for simple induction of stroke. Therefore, an aberrant clot is as effect as a natural clot for the purpose of prey capture. Thus, the selection pressure is for speed of action, not how well-ordered the fibrin clot is. Thus, if an aberrant thrombin form cleaves fibrinogen faster than the endogenous form, the selection pressure will be for venom molecules specific for the cleavage site that activate prothrombin into the fast-acting aberrant form, even if the abberant thrombin in turn produces abberant fibrin clots. Such clots would still be strong enough to produce a stroke in a prey animal, with such prey subjugation being the primary evolutionary selection force. Therefore, as the clots are strong enough to satisfy this selection pressure, speed of clot formation is the function selected for.

It is known that vertebrate blood clotting is more complex in mammals than it is in earlier diverging vertebrates, involving more haemostasis-related genes than other vertebrates [70,71]. For example, toad plasma lacks the homolog of the human factor XI, and, with the exception of fibrinogen, all other coagulation factors are found in lower concentrations compared to human blood [70]. In contrast, toad plasma has a relatively high concentration of antifibrinolytic agents [72]. Birds are deficient in Factor XII, consequently normal avian blood coagulates very slowly in some testing conditions with clotting times that can exceed 70 min [70,72]. An interesting finding of our study was that the human thrombin positive control was not able to clot toad plasma (Figure 6). However, the human FXa was able to clot this plasma, but inducing weaker clots than the venoms. In this case, the differences found could involve genetic variations underlying the structure of coagulation factors among the anurans [73,74].

Reflective of these differences was the convergence between speed and clot strength in amphibian plasma for the *B. atrox* (Santarém, PA), which predates heavily upon amphibians in the floodplains that it inhabits. This same prey item has greater escape potential and is the least trackable (in water) than mammalian prey which are less able to escape and are easier to track (on land), and therefore there is an extreme selection pressure for speed of action. Thus, the *B. atrox* (Santarém, PA) venom is evolving under two novel selection pressures (prey specialization and prey escape), relative to the other *Bothrops* venoms in this study. *B. atrox* (Santarém, PA) is a specialist for a prey item that has a different clot-inducing landscape than that of avian or mammalian plasmas, therefore exerting a selection pressure for toxin specialisation. Reflective of the changes in clotting biochemistry with the amphibian plasma relative to the avian and human plasmas, the thrombin control was inactive against this plasma, while the Factor Xa control performed poorly. *B. atrox* (Santarém, PA), however, was substantially faster at clotting avian and human plasmas than other *Bothrops* venoms while also producing the strongest clots in the amphibian plasma and being the fastest on this plasma too. Therefore, this venom displays the greatest adaptive flexibility of all the venoms in this study. Thus, the two functions (speed of action versus clot strength), which appear to be mutually exclusive selection pressures in other plasma for all the *Bothrops* venoms, appear to be overlapping in the *B. atrox* (Santarém, PA) venom. This is indicative of nuances in the amphibian endogenous thrombin in that the form which most quickly converts fibrinogen to fibrin clots is also the most biochemically efficient in producing strong clots. In contrast, for the avian and human plasmas the results are indicative of mutually exclusive forms of thrombin products generated by the venom, in which the thrombin which have the fast rate of action are not the same forms which produce the best-order, strongest fibrin network. Also noteworthy is the *B. atrox* venom from São Bento, which entirely lacked activity on amphibian plasma. This finding likely reflects a drier habitat and a lower dependence on anurans within the diet, as the São Bento region is located in a transition zone between the Amazon forest and the Brazilian cerrado—a different biome. These interesting patterns should be the subject of follow up research examining the sites of prothrombin which are targeted for cleavage and also the architecture of the fibrin clots produced by the different thrombin forms. Such variations between different venoms on the same plasma or the same venom on different plasmas will be a fascinating area of future research.

Together the results in this study contribute to the body of knowledge regarding prey specific venom effects of coagulotoxins, which have been noted previously for diapsids (bird/reptile) versus synapsid (mammal) plasmas for *B. neuwiedi* [49]. Procoagulant variation has been noted between taxon for other venoms against a range of mammals [75]. However, in other cases taxon-specific procoagulant effects have not evolved due to constraints imposed by the target site, where mutant forms of endogenous Factor Xa retains endogenous Factor Xa’s prothrombin cleavage site as a consequence of the historical contingency of this toxin class being formed by venom expressed endogenous clotting enzymes with structural adaptive limitations [5,69]. Taxon-specific effects have been noted for neurotoxic actions by colubrid [76,77,78,79] and elapid [80,81,82] venoms. Prey-specific variation in overall lethality has also been noted for viperid venoms but was not attributed to a specific venom function [83]. In addition, ontogenetic variation in venom composition is also an evolutionary feature, such as the age-related shift in *Pseudonaja* species (brown snakes) from neurotoxic venoms in lizard-specialist neonates, to procoagulant venoms in mammal-specialist adult snakes [67,84].

In summary, this multifaceted study documented variations in coagulotoxic effects and differential antivenom efficacy within the *Bothrops* genus, a snake group of extreme medical importance in South America. Our results revealed new information about the effects of underlying biochemical variations in venoms, as well as the evolutionary pressures that could be shaping venoms of *B. atrox* populations in different locations from the Brazilian Amazon. When tested under physiologically relevant conditions, the venoms in this study demonstrated procoagulant potencies exceeding that of other strongly procoagulant genera (tested under identical conditions), such as *Echis* within the Viperidae family [8], *Notechis* within the Elapidae family [5], and *Atractaspis* within the Lamprophiidae family [6]. Therefore, the findings here reported bring important insights about issues involving functional diversity of snake venoms, human envenomations, antivenom efficacy, and also prey-specific effects, which may be of interest to a broad audience, including clinicians and ecologists.

## 4. Materials and Methods

### 4.1. Venoms

*B. atrox* venom samples were obtained from adult snakes (males and females), captured from nature (cities of Santarém (in a várzea floodplain habitat) and Belterra (in an upland forest ‘terra firme’ habitat), State of Pará—under SISBio license 32098-1) and kept in captivity for two years. *B. atrox* venom samples, which were obtained from snakes captured at São Bento—State of Maranhão, and Presidente Figueiredo—State of Amazonas, and kept in captivity for several years, were provided by Laboratório de Herpetologia and Museu Biológico from Instituto Butantan, respectivally. To account for individual variation in venom composition, equal amounts of individual venoms were added to prepare four pools of venoms representing each area, as follows: Santarém *n* = 9, Belterra *n* = 10, São Bento *n* = 10, Presidente Figueiredo *n* = 13. The *B. jararaca* and *B. neuwiedi* venoms were provided by Herpetarium from Instituto Butantan (specific collection localities not recorded). The access to the Brazilian genetic heritage was registered in the SISGEN platform under the number ABEC205. All venom samples were exported under license from Brazil (export permit: 17BR026238/DF) to Australia (import permit: IP 15016115).

### 4.2. Antivenom

The polyvalent Bothrops antivenom (batch: 1305077, ED: 05/16), produced by Instituto Butantan, Brazil, from horses immunised with a pool containing venoms of five Bothrops species:*B. jararaca*, (50%), *B. neuwiedi* (12.5%), *B. jararacussu* (12.5%), *B. alternatus* (12.5%), and *B. moojeni* (12.5%). The antivenom solution consists of soluble IgG F(ab’)^2^ fragments and the stipulated potency by manufacturer is: 1 mL neutralises 5 mg of the Bothrops venom of reference. For use in this study’s neutralization assays, the antivenom was centrifuged (12,000 RCF, 10 min at 4 °C), the supernatant removed, filtered (0.45 nm filter), aliquoted (1 mL tubes), and stored at 4 °C until use.

### 4.3. Plasmas

Human plasma was obtained from the Australian Red Cross (Research agreement #18-03QLD-09; University of Queensland Human Ethics Committee Approval #2016000256). Avian (domestic chicken) and amphibian (cane toad, Rhinella marina) plasma were obtained under the University of Queensland Animal Ethics approval SBS/019/14/ARC. All plasma was prepared as 3.2% citrated stock and then aliquoted into 1 mL quantities, which were snap frozen in liquid nitrogen, and stored at −80 °C until needed, at which time an aliquot was defrosted by placing into a 37 °C water bath for 10 min. All venom and plasma work was undertaken under University of Queensland Biosafety Approval #IBC134BSBS2015.

### 4.4. Coagulation Analyses

Coagulation analyses using a Stago STA-R Max coagulation analysis robot were undertaken as previously described, with the antivenom diluted to 5% for use in those protocols [5,6,8,9].

### 4.5. Factor X and Prothrombin Activation Activity

In the activation assays, venom stock solutions at 1 mg/mL (in 50% deionised H_2_O: 50% glycerol) were diluted with OK buffer (Owren Koller Buffer, Stago, Asnières sur Seine, France), 40 μL of venom into 360 μL of OK buffer. Following, 50 μL of the samples, 50 μL of CaCl_2_ (25 mM), 50 μL phospholipid (cephalin prepared from rabbit cerebral tissue from C.K Prest standard kit, Stago, Asnières sur Seine, France, solubilised in OK buffer) and 25 μL of OK buffer were added automatically into cuvettes. The mixture was incubated (2 min, 37 °C) before adding 75 μL of a solution containing the colorimetric substrate (Catalog 00311, Stago, Asnières sur Seine, France) and FX (at 0.01 μg/μL) or Prothrombin (at 0.1 μg/μL), with a final volume of 250 μL in the cuvette. Changes in the optical density (OD) were measured each second, for 300 s, using a STA-R Max^®^ automated analyser (Stago, Asnières sur Seine, France). The activation rates of FX or prothrombin were calculated considering the OD variation corresponding to cleavage of the substrate by FXa or Thrombin, after the activation of the zymogens, in relation to direct cleavage of substrate by the venoms in the absence of the zymogen. Human FXa or thrombin as samples (instead of venom) were used as positive controls to ensure functionality of the substrate. Each venom, analysed in the same conditions, without the presence of the zymogens, was used as its own negative control to ensure no direct cleavage of the substrate by the venom. The results represent the mean ± SD of experiments performed in triplicate.

To investigate the antivenom efficacy against the venom components able to activate FX or prothrombin, all test conditions from the colorimetric cleavage assays were replicated (Section 4.5), except that 25 μL of antivenom, from a working solution (50 μL of the reconstituted antivenom in 950 μL OK buffer), was used in place of 25 μL of OK buffer. Then, reaction mixture was incubated (2 min, 37 °C), before adding 75 μL of the solution containing the colorimetric substrate and FX (at 0.01 μg/μL) or prothrombin (at 0.1 μg/μL), in a final volume of 250 μL/cuvette, and the cleavage of the substrate was measured each second for 300 s in the automated analyser (STA-R Max^®^). The antivenom efficacy was calculated comparing the activation rates in the presence vs. the absence of antivenom, and the results represent the mean ± SD of experiments performed in triplicate.

### 4.6. Thromboelastography and Fibrinogenolytic Analyses

Were undertaken as previously described [6,85].

### 4.7. Cleavage of Fibrinogen

Fibrinogen cleavage studies were conducted in 1mm SDS-PAGE gels (12%), as previously described [85,86,87].

### 4.8. Statistical Analyses

All dose-response curves, as well as cofactor dependency tests, were conducted in triplicate and we present the results as mean and standard deviation. Data were analysed using Prism 7.0 software (GraphPad Software Inc., La Jolla, CA, USA, version 7, 2017, La Jolla, CA, USA). Tests for correlation were undertaken using the R-studio Pearson correlation function: step 1 tox<-read.csv(file.choose(),header=T); step 2 cor.test(tox$var1,tox$var2).
